# Supplementary material for: Biopsychosocial Aspects in Individuals with Acute and Chronic Rotator Cuff Related Shoulder Pain: Classification Based on a Decision Tree Analysis
Source: Diagnostics (Basel). 2020 Nov 10;10(11):928. doi: 10.3390/diagnostics10110928 (PMC7696199; doi:10.3390/diagnostics10110928)
Supplement: Supplementary file 1 [file diagnostics-10-00928-s001.pdf]

Supplementary Material. Overview of the different methods used with their specific outcomes.

**Outcome**	**Method**	**Units/Result options**
Sociodemographic aspects		
Age	Interview	Range: 18-80 years
Sex	Interview	Male/Female
Educational level	Interview	Incomplete elementary school<br>Elementary school<br>High school<br>University education
Marital status	Interview	Single<br>Married<br>Divorced<br>Widowed
Dominance	Interview	Right /Left
Employment status	Interview	Active/Retired
Type of occupational demand	Interview	Physical<br>Psychological<br>Both
Presence of upper limb repetitive movement	Interview	Yes/No
Physical activity frequency	Interview	Days/week
Smoking status	Interview	Yes/No
Biological aspects related to general clinical health status		
Presence of any kind of hypersensitivity	Interview	Yes/No
Self-reported presence of any other frequent symptoms	Interview	Fatigue<br>Concentration difficulties<br>Sleep disturbance<br>Swollen feeling<br>Tingling<br>Numbness
Presence of any other pain previous to shoulder pain	Interview	Yes/No
Pain intensity of the previous most painful complaint besides the shoulder pain	Numerical Pain Rating Scale, NPRS	Range: 0-10
Central sensitization degree	Brazilian version of the Central Sensitization Inventory (CSI-Part A)	Range: 0-100
Biological aspects related to shoulder clinical condition		
Unilateral or bilateral pain	Interview	Dominant<br>Non-dominant<br>Bilateral
Duration of symptoms	Interview	Number of Months
Pain intensity during arm elevation	Physical exam	Range: 0-10
Abduction and external rotation painful range of motion (ROM) or angular onset of pain	Inclinometry	Degrees
Presence of scapular dyskinesis	Dynamic scapular dyskinesis test	Present/Absent
Influence of scapular motion on shoulder disorder	Scapular assistance test	Positive/Negative

Irritability of shoulder structures	Tests: Neer Jobe, Hawkins-Kennedy, resisted shoulder lateral rotation, Speed, Gerber, Yocum, cross-body aduction, acromion clavicular pressure, Belly Press	Number of positive tests
Disability of the upper limbs	Brazilian version of the Disabilities of the Arm, Shoulder and Hand Questionnaire (DASH)	Range: 0-100
Biological aspects related to sensory function		
Tactile acuity	Two-point discrimination threshold (TPDT), anterior and posterior shoulder regions	Range: 0-60mm
Body image performance	Left/right judgment task (LRJT)	Percentage of images correctly judged<br><br>Response time as the time in seconds dispended to decide whether each picture showed a right or left shoulder
Minimum amount of pressure that provoked the first onset of pain	Pressure pain threshold (PPT) at a bone surface (PPT-acromion) and a muscle belly region (PPT-deltoid) of the affected (or most affected) side, as well as at a remote region (PPT-tibialis anterior)	KPa
Temporal summation (TS)	Repetition of PPT value at the same region (TS-acromion, TS-deltoid and TS-tibialis anterior)	Range: 0-10
Capacity of the central system to inhibit pain through the conditioned pain modulation (CPM) model	Conditioned pain modulation	PPT-acromion percentage changes from baseline to during cold pressor test (CPM-during) and from baseline to after cold pressor test (CPM-after)
Psychosocial aspects		
Fear-avoidance	Brazilian version of Fear-Avoidance Beliefs Questionnaire (FABQ-Br)	Range: 0-96
Kinesiophobia	Tampa Scale for Kinesiophobia (TSK)	Range: 0-68
Catastrophic thoughts about pain	Brazilian version of the Pain Catastrophizing Scale (PCS)	Range: 0-52
Self-efficacy and self-ability to cope with pain, physical activity and symptoms	Chronic Pain Self-Efficacy Scale (CPSS)	Range: 200-2000
Negative emotional aspects of each domain: depression, anxiety and stress	Depression, Anxiety and Stress Scale-21 (DASS-21)	Range: 0-63
Quality of life	Brazilian version of Euroqol instrument (EQ-5D)	Range: 0-1
